# The evolution of humoral immune responses to past and novel influenza virus strains gives evidence for antigenic seniority

**DOI:** 10.3389/fimmu.2022.987984

**Published:** 2022-09-02

**Authors:** Federica Sicca, Eleni Sakorafa, Anouk de Jonge, Jacqueline de Vries-Idema, Fan Zhou, Rebecca Jane Cox, Anke Huckriede

**Affiliations:** ^1^ Department of Medical Microbiology, University of Groningen, University Medical Center Groningen, Groningen, Netherlands; ^2^ Influenza Centre, Department of Clinical Science, University of Bergen, Bergen, Norway; ^3^ Department Microbiology, Haukeland University Hospital, Bergen, Norway

**Keywords:** influenza virus, antibodies, strain-specificity, longitudinal cohort, H1N1pdm09

## Abstract

The high genetic and antigenic variability of influenza virus and the repeated exposures of individuals to the virus over time account for the human immune responses toward this pathogen to continuously evolve during the lifespan of an individual. Influenza-specific immune memory to past strains has been shown to affect the immune responses to subsequent influenza strains and in turn to be changed itself through the new virus encounter. However, exactly how and to what extent this happens remains unclear. Here we studied pre-existing immunity against influenza A virus (IAV) by assessing IAV binding (IgG), neutralizing, and neuraminidase-specific antibodies to 5 different IAV strains in 180 subjects from 3 different age cohorts, adolescents, adults, and elderly, over a 5-year time span. In each age cohort, the highest neutralizing antibody titers were seen for a virus strain that circulated early in their life but the highest increase in titer was found for the most recent virus strains. In contrast, the highest IgG titers were seen against recent virus strains but the biggest increase in titer occurred against older strains. Significant increases in neutralizing antibody titers against a newly encountered virus strain were observed in all age cohorts demonstrating that pre-existing immunity did not hamper antibody induction. Our results indicate that the evolution of influenza-specific humoral immunity differs for rather cross-reactive virus-binding antibodies and more strain-specific neutralizing antibodies. Nevertheless, in general, our observations lend support to the antigenic seniority theory according to which the antibody response to influenza is broadened with each virus encounter, with the earliest encountered strain taking in the most senior and thus dominant position.

## Introduction

Influenza virus used to and probably will again represent a major burden for society. Until 2020, annual influenza epidemics caused ~1 billion infections, 3 to 5 million cases of severe illness, and about 290 000 to 650 000 respiratory deaths (1). Of the 4 influenza virus types A, B, C and D, type A is of particular importance as it is the only type with pandemic potential (2, 3). Influenza A viruses (IAV) are subtyped based on the sequence and the antigenic distance of the surface proteins hemagglutinin (HA) and neuraminidase (NA) proteins (2–4). So far, 18 HA and 11 NA subtypes have been identified, the combination of which defines the different virus subtypes. Currently, H1N1pdm09 and H3N2 subtypes are co-circulating and cause seasonal epidemics (2, 3). Within each subtype different virus strains are distinguished based on their exact antigenic properties.

Every year new IAV strains emerge because of point mutations in the viral HA and NA genes. This phenomenon, called “antigenic drift”, allows partial immune escape and thus sustains the permanent circulation of influenza viruses associated with yearly epidemics. Moreover, “reassortment” of viral genome segments during co-infection of a host with two different IAV strains every now and then brings about a completely new strain, with novel HA and/or NA molecules derived from antigenically diverse strains of influenza virus (5). This phenomenon called “antigenic shift” accounts for occasional influenza pandemics since the newly emerging virus meets a population which is naïve to the novel HA (and NA).

The ever-changing nature of IAV and the repeated exposures to the virus cause the human immune responses towards this pathogen to evolve during the lifespan of an individual (2, 6, 7). Understanding how immune history affects the production of different types of antibodies in terms of their antigenic target and mechanisms of action is crucial for improving the current vaccination strategies and accordingly has been a subject of research for decades.

In the early 1950s, Thomas Francis Jr. and colleagues formulated the “original antigenic sin” (OAS) theory (8). According to this theory, the first exposure to IAV during childhood leaves an immunological ‘imprint’. Later encounter of antigenically different IAV strains would cause a boost of the antibody response to the imprinting virus strain rather than induction of *de novo* responses to the new strain resulting in a low-affinity response to the new viral antigens (8–13). Recently, Lessler et al. proposed a refined version of the OAS theory, termed “antigenic seniority” (AS) theory (6, 14–17). According to this theory, each encounter of an IAV strain would elicit antibodies to the new strain but would also boost the responses to all the previously encountered strains. Consequently, as the antibodies specific for the most senior strains would be boosted most often, they would be most prevalent, followed by progressively lower levels of antibodies specific for increasingly recent influenza strains. In this model, the response to the new virus strain is not necessarily impaired and we would witness over time a broadening of the influenza-specific antibody repertoire (2, 6, 9, 10, 18, 19).

Firm data confirming or rejecting either theory is scarce because of the paucity of longitudinal studies focusing on the evolution of the influenza specific antibody repertoire in an individual over time (9, 17). In the current study, we therefore investigated in sequential samples from adolescents, adults and elderly how IAV-specific antibody titers, acquired by past IAV exposures, differ among different age groups, how they evolve over time in these groups, and how pre-existing antibodies impact the immune response to a novel pandemic influenza virus strain. To address these questions, we exploited Lifelines, a large, 3 generation prospective cohort study, based in Groningen, The Netherlands (https://www.lifelines.nl/researcher). From the Lifelines biobank we selected sequential serum samples, taken with 5-year interval, from 180 subjects, 60 per age cohort. For the adults and the elderly cohort these samples were taken in spring 2009 and in 2014, thus before and after the emergence of the 2009 pandemic. This allowed us to study changes in IAV-specific immune responses imposed by the pandemic influenza virus strain. We determined total influenza-specific immunoglobulin (IgG) antibody titers and virus-neutralizing (VN) antibody titers to 5 different IAV strains which circulated in the population in different periods spanning the entire life time of the study groups. In addition, we assessed antibodies specific for neuraminidase (NA) of H1N1pdm09.

Our study shows that VN antibody levels displayed clear birth year-dependent differences, with the absolute highest titers found against strains encountered early in life in all three age cohorts. Yet, encounter of a novel influenza virus strain resulted in a significant increase of titers to the novel but not to previously encountered strains. In contrast, IgG titers were highest against the most recently encountered strains in all age groups and were mainly boosted against past strains upon encounter of a novel influenza virus strain. Finally, NA-inhibiting antibodies also showed clear birth year-dependent differences and were effectively induced by the encounter of a novel influenza virus strain. Taken together, the pattern of humoral immune memory that we observed here was roughly in line with the AS theory.

## Material and methods

### Serum samples

**Figure 1:**
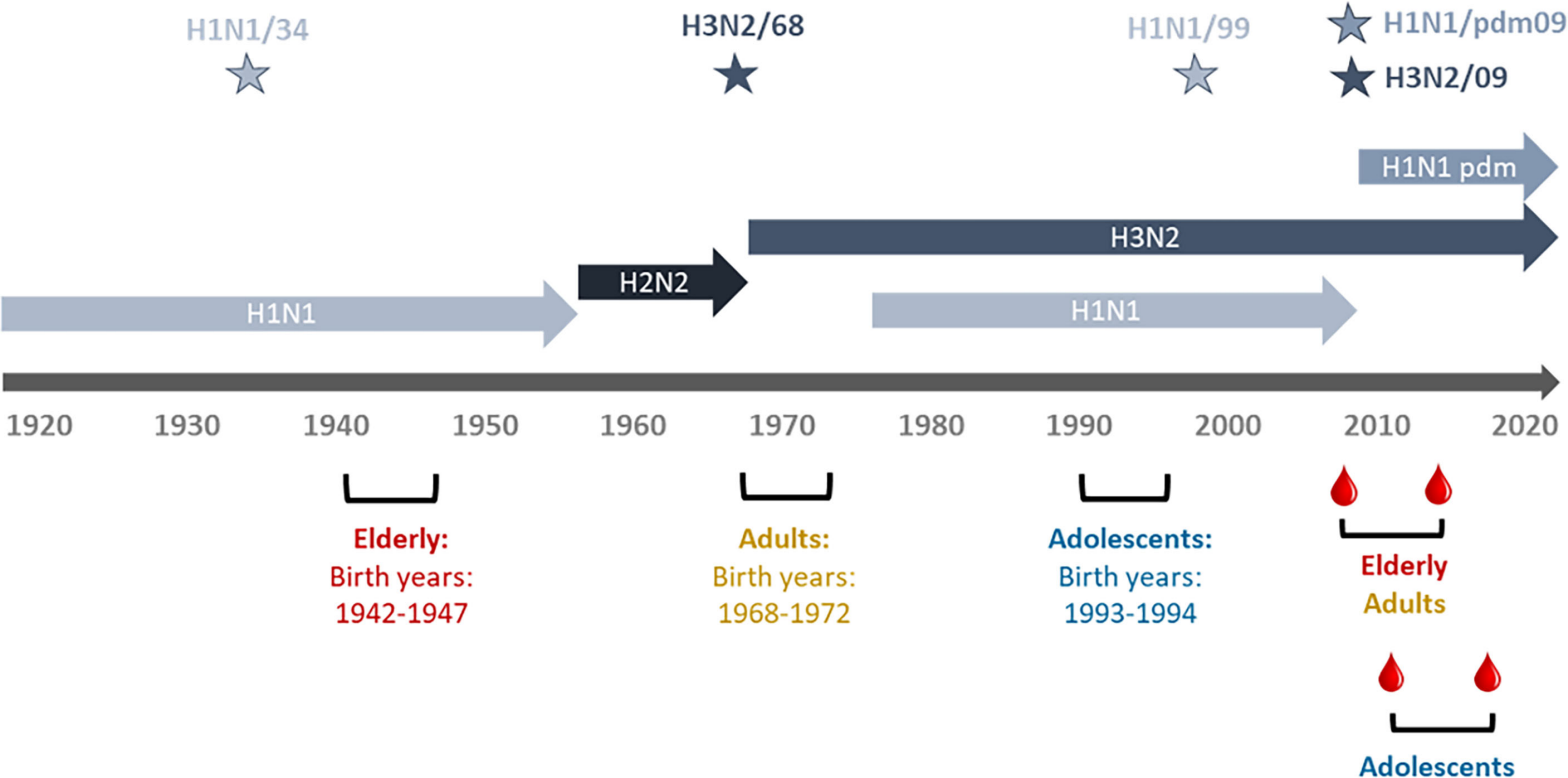
Timeline of IAV circulation, cohort birth dates, and sampling period. Depicted are the different influenza virus subtypes and the periods during which they were circulating. Stars denote the virus strains used in this study (H1N1/34: A/Puerto Rico/8/34, H3N2/68: A/Aichi/1/68, H1N1/99: A/New Caledonia/20/99, H1H1pdm09: A/California/7/2009, H3N2/09: A/Perth/16/2009. The birth periods of the study cohorts are shown in red for the elderly, in green for the adults and in ochre brown for the adolescents. Dates of blood sampling are indicated for the adults and elderly and for the adolescents.

Serum samples were collected from subjects enrolled in the “Lifelines Biobank” (Groningen, Netherlands) (20). Lifelines is a multi-disciplinary prospective population-based cohort study examining in a unique three-generation design the health and health-related behaviours of > 167,000 persons living in the north of The Netherlands. Blood samples were collected from 180 participants at two different time points, with a time interval of 5 years in between; referred to as “assessment 1” (a1) and “assessment 2” (a2) in the following. The participants belonged to three different age cohorts (**Figure 1**; **Table 1**). At the time of a1, participants of the elderly cohort were 62-67 years old (years of birth: 1942-1947), participants of the adult cohort aged 37-41 (years of birth: 1968- 1972) and participants of the adolescent cohort aged 17-18 (years of birth: 1993-1994). The a1 for adults and elderly participants happened in 2009, while a1 for adolescents happened in 2011-12. Assessment 2 for all cohorts happened approximately 5 years after a1, therefore in 2014 for adults and elderly and in 2015-17 for adolescents. The gender of participants was known and a 50% ratio of female/male could be achieved in all cohorts except the adolescent cohort, for which the ratio was disproportionate with 75% of females. In order to exclude, as much as possible, vaccinated subjects, we selected for this study only subjects not affected by asthma, cancer or diabetes for whom vaccination is recommended in the Netherlands. Vaccination and infection history of the subjects regarding influenza virus was unknown.

**Table 1:** Description of study population and assessments.

	No	Male/female	Years of birth	Assessment 1	Assessment 2
**Adolescents**	60	15/45	1993-1994	2011-2012	2015-2017
**Adults**	60	30/30	1968-1972	2009	2014
**Elderly**	60	30/30	1942-1947	2009	2014

### Influenza virus strains

The influenza virus strains that were used in this study for ELISA assays and VN assays were the pandemic strains A/California/7/2009 (H1N1 pdm09) and A/Aichi/1/68 (H3N2/68) and the seasonal strains A/Puerto Rico/8/34 (H1N1/34), A/New Caledonia/20/99 (H1N1/99), and A/Perth/16/2009 (H3N2/09). We used the recombinant H7N1 strain NIBRG127 for the ELLA assay. All virus strains were obtained from the National Institute for Biological Standards and Controls, Potters Bar, UK, and were propagated in embryonated chicken eggs.

### Enzyme-linked immunosorbent assay (ELISA)

ELISA high binding capacity plates were coated with 0.3 μg of whole inactivated influenza virus (WIV) in 100 μl of coating buffer (0.05 M carbonate-bicarbonate pH 9.6-9.8) per well. After a one-hour incubation at 37°C, the plates were washed with coating buffer once and blocked with 2.5% milk solution in coating buffer (200 μl/well) for 45 minutes at 37°C. The plates were washed once with coating buffer and twice with PBS/Tween (PBS with 0.05% Tween-20). The serum samples were added in the wells and serial dilutions were performed so that every well ended up containing 100 μl of serum per well. Incubation for 1,5 hours at 37°C followed, after which the plates were washed three times with PBS/Tween. The plates were incubated for 1 hour at 37°C in the dark with horseradish peroxidase (HRP)-labeled mouse anti-human IgG Fc secondary antibody (Thermo Fisher Scientific Catalog # 05-4220) diluted 1:1500 in PBS/Tween, and then washed three times with PBS/Tween and once with PBS. 100 μl of SIGMAFAST™ OPD (o-Phenylenediamine dihydrochloride) in phosphate buffer was added to the plates according to manufacturer’s instructions. After 30 mins incubation time (in the dark) the reaction was stopped with 50 μl/well of H2SO4. Optical density (OD) of the ELISA plates was read in an ELISA reader at 490 nm.

In order to calculate the total IgG antibody titers of the serum samples, the average optical density of the cell controls was measured (mean OD), the standard deviation (SDEV) of the cell control was determined and finally the cut-off for the presence of IgG influenza specific antibodies was calculated with the following formula: mean OD+ (SDEV x f), in which f equals a standard deviation multiplier based on the number of control samples and chosen confidence level. Here, the number of control samples was 8 and the confidence level was 99.9%, which determines that f = 5.076 (21).

### Virus neutralization (VN) assay

Serum samples (1:10 diluted in 100 μl) were added to the first column of 96-well plates and serially diluted two-fold across the plate. 50 μl of virus solution (working dilution containing 100 TCID_50_/50 μl) were added to all the wells. The control wells were designed as follows: 100 μl of medium alone were added in the wells that served as “cell controls”, while 50 μl of medium and 50 μl of virus (working dilution containing 100 TCID_50_/50 μl) were added to the wells that served as “virus control”. The plates were incubated at 37°C for one hour. At this point, 150 000 cells/ml of MDCK cells (Madin-Darby canine kidney) were added in every well. The plates were placed in an incubator at 37°C for 20 hours. The plates were washed once with sterile PBS (200 μl/well), and then incubated for 10-12 mins at room temperature with 100 μl/well of cold 80% Acetone in sterile PBS (serving as a fixative). The acetone was then discarded and the plates were left to air-dry for 5 mins. The plates were washed once with wash buffer (PBS with 0.3% Tween-20), blocked with blocking buffer (5% milk in wash buffer) for 1 hour at room temperature, and washed 3 times with wash buffer. 100 μl/well of the primary antibody (anti-influenza A nucleoprotein-specific antibody, Merck MAB8257) diluted 1:3000 in blocking buffer was added to the wells and the plates were incubated for 1 hour at room temperature. At this point the plates were washed three times with wash buffer, then 100 μl/well of the secondary antibody (HRP-labelled goat anti-mouse IgG (H+L), Thermo Fisher Scientific Catalog # 32430) diluted 1:200 in blocking buffer was added to the plates. The plates were incubated for one hour at room temperature in the dark. The plates were washed 3 times with wash buffer and then 200 μl of freshly prepared substrate (o-Phenylenediamine dihydrochloride, Sigma Aldrich) was added in every well following manufacturer’s instruction. The reaction was stopped after 30 minutes of incubation at room temperature in the dark by using sulfuric acid (50 μl/well). The optical density (OD) was read using a spectrophotometer (wavelength= 490 nm).

The OD value X which represents the cut-off for virus neutralization was calculated as follows:


X=(Average OD of VC wells+Average OD of CC wells)/2

where the cell control (CC) consisted of non-infected cells and the virus control (VC) consisted of infected cells without addition of serum. All wells with an OD (490 nm) below or equal to ‘‘X” were considered positive for neutralization activity. The VN titer was determined as the reciprocal of the highest serum dilution with an OD ≤X.

### Enzyme-linked lectin assay (ELLA)

The ELLA was used to measure antibodies inhibiting the ability of NA to cleave sialic acid (22). We used NIBRG127 from NIBSC, an H7N1 virus containing the HA from an equine influenza virus strain and NA from A/California/07/09 and grew it under BSL-2+ conditions in line with Dutch legislation. Ninety-six well flat bottom Maxisorb plates (VWR, USA) were coated with 100μl of coating buffer (1x KPL coating buffer, SeraCare, Milford, USA) containing 25 μg/ml fetuin (Sigma-Aldrich, Zwijndrecht, The Netherlands). Fetuin-coated plates were sealed with a plate sealer and stored at 2-8°C for at least 18h or until needed (for a maximum of 2 months). Fetuin-coated plates were then washed 3 times with the wash buffer (1xPBS containing 0.05% Tween), after which the plates were blotted onto absorbent paper to remove excess fluid. Heat-inactivated (56°C for 45–60min) serially diluted serum, starting from 1:10 initial dilution, and H7N1 virus were added to the plates and incubated at 37°C for 16–18 hours.

The virus was diluted to a titer giving an OD corresponding to 90% NA activity. Next, 100 μl of 1 mg/ml conjugated peanut agglutinin (PNA)-horseradish peroxidase (HRP, Sigma-Aldrich) dissolved in conjugate diluent (1x PBS containing 1% BSA) was added to all wells, after which the plates were left for 2hrs at room temperature in the dark. Plates were developed by addition of 100 μl of o-phenylenediamine dihydochloride (OPD, Sigma-Aldrich, USA) in citrate buffer to all wells following manufacturer’s instructions. After 10 min incubation at room temperature in the dark, the reaction was stopped with 100 μl 1M sulphuric acid. The plates were read with a microplate reader by spectrophotometry at 490 nm wavelength. The anti-NA antibody titers (reported as 50% inhibition concentration, IC50) of the serum samples were calculated as the reciprocal dilution of serum which gave OD values equal to 50% of total OD [(OD virus control + OD blank)/2)] in four-parameter non-linear regression analysis using GraphPad Prism.

### Graph design and statistical analysis

All graphs shown in this study were generated with GraphPad Prism (version 8.0.0 for Windows, GraphPad Software, San Diego, California USA, www.graphpad.com). Statistical significance for GraphPad is displayed as: *=P ≤ 0.05; **=P ≤ 0.01; ***=P ≤ 0.001; ****=P ≤ 0.0001.

## Results

### Neutralizing antibody responses but not IgG responses are governed by age- and strain-specific differences

To understand how sequential exposure to different IAV virus strains can shape the humoral immune response we made use of serum samples collected from participants of the Groningen-based cohort study Lifelines. This cohort study follows 167 000 inhabitants of the northern regions of the Netherlands, and collects oral information, blood and urine samples every 5 years. For our study, we selected 60 elderly, 60 adults and 60 adolescent individuals (**Figure 1**). We determined VN titers and IgG antibody titers against five different influenza virus strains, H1N1/34, H3N2/68, H1N1/99, H3N2/09 and H1N1pdm09, in serum samples collected from the selected individuals at two timepoints (a1 and a2) 5 years apart. These strains were chosen according to the following criteria. We selected at least one virus strain per age cohort which could represent the imprinting strain or a drift variant of the imprinting strain for the given age group. H1N1/34, for the elderly, H3N2/68 for adults, and H1N1/99 for adolescents. We also included H3N2/09 and H1N1pdm09 to analyse the immune response of the subjects to strains circulating close to the blood sampling moments, including the newly emerged influenza virus pandemic strain. Virus-binding antibodies, virus-neutralizing antibodies and NA-specific antibodies were determined by ELISA, VN assay, and ELLA, respectively.

**Figure 2:**
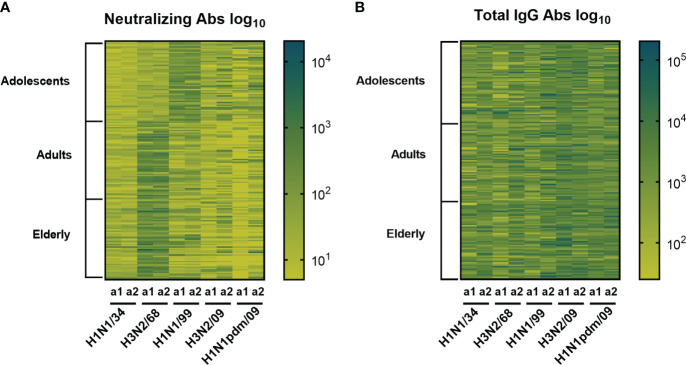
Heatmaps displaying influenza specific and neutralizing antibody titers and total IgG titers. Sera obtained from 180 subjects (belonging to three age cohorts: adolescents, adults, elderly) taken at 2 different time points (a1, a2) were assessed for virus neutralizing **(A)** and virus binding antibodies **(B)** against 5 historic influenza virus strains indicated on the X axis. Each column represents responses (log_10_ antibody titers) to one virus strain at one sampling moment (a1 or a2). Each row represents the responses of one individual. The darker the color the higher the antibody titer.

Heatmaps summarizing the results from the VN and ELISA assays for each individual and each strain are depicted in **Figure 2**. The heatmap to the left demonstrates that VN antibody titers differed according to the strain tested and the age group the subjects belonged to and were highest against strains circulating in the past. In contrast, total IgG antibody titers did not show age-specific differences in the heatmap and in all age groups the highest titers were seen against strains that circulated recently with respect to the blood sampling moments (**Figure 2**).

### Neutralizing antibody titers reflect early virus encounters while IgG titers reflect recent virus encounters

**Figure 3:**
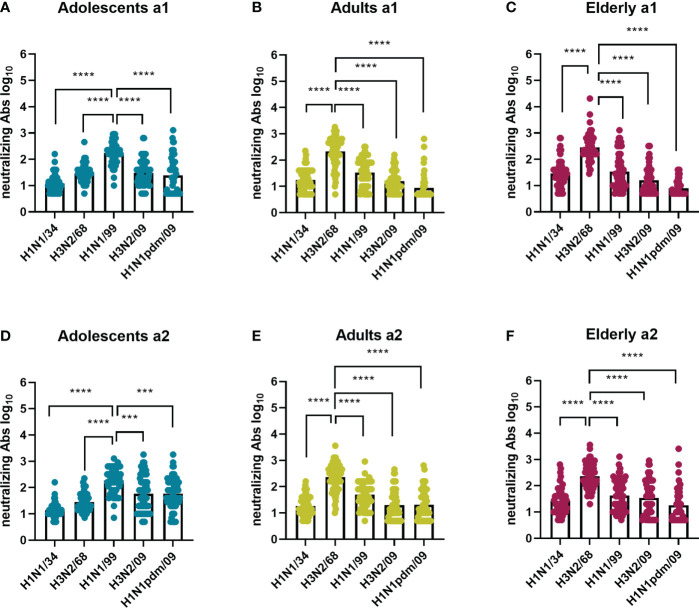
Influenza virus strain-related differences in neutralizing antibody titers per age cohort. Sera collected at 2 time points (a1 and a2) from adolescents **(A, D)**, adults **(B, E)**, and elderly **(C, F)**, 60 per age cohort, were assessed for virus neutralizing antibodies against 5 historic influenza virus strains: H1N1/34, H3N2/68, H1N1/99, H3N2/09, H1N1 pdm09. For each assessment and age cohort data were analyzed using Friedman test followed by Dunn’s post-hoc test for multiple comparison. ***P≤0.001; ****P≤0.0001.

In order to detect possible age-dependent strain preferences we plotted the results for each of the virus strains per age cohort. Our data revealed that in both assessments VN antibody titers for adolescents were highest against H1N1/99 and for adults against H3N2/68, thus the respective virus strains these age groups encountered first in life (**Figure 3**). VN titers for the elderly were also highest against H3N2/68 and not against H1N1/34 which would have been more closely related to their imprinting strain. Not only were the titers highest against strains encountered early in life but also the number of individuals in the respective age groups with a titer ≥80, considered as putatively protective, was highest against those strains, ≥50 out of 60 as opposed to 0-30 out of 60 for other strains (**Supplementary Figures 1A–C**).

**Figure 4:**
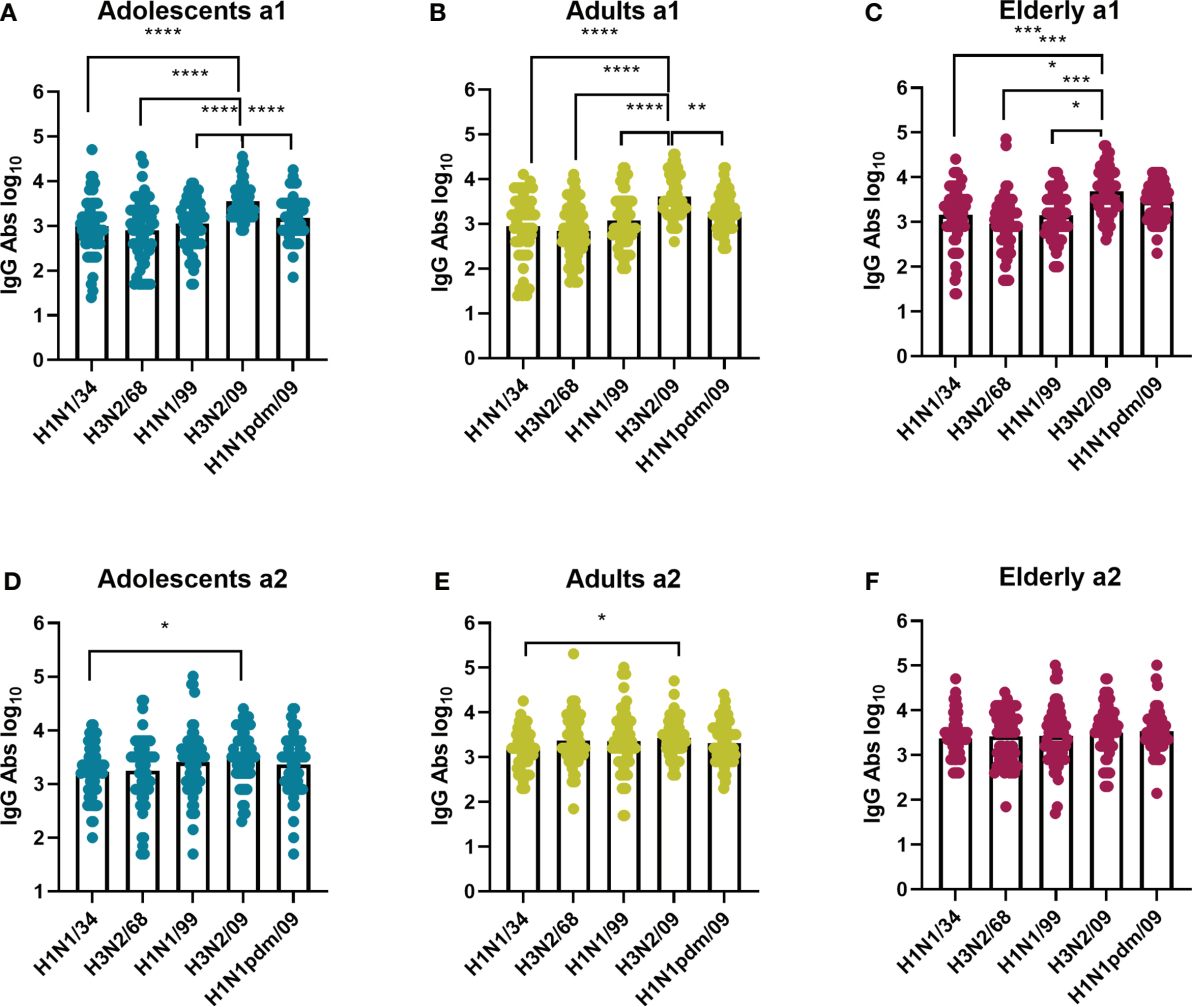
Influenza virus strain-related differences in IgG antibodies titers per age cohort. The sera from adolescents **(A, D)**, adults **(B, E)**, and elderly **(C, F)** taken at a1 and a2 were assessed by ELISA for total IgG antibodies against the same virus strains as in **Figure 2**, indicated on the X axis. Data were analyzed using Friedman test followed by Dunn’s post-hoc test for multiple comparison. *P≤0.05; **P≤0.01; ***P≤0.001; ****P≤0.0001.

While there was a clear age-related difference in strain preference with regard to VN antibody titers this was not the case for IgG titers (**Figure 4**). In a1, IgG antibody titers were moderately but significantly higher against H3N2/09 than against all other virus strains for all the age groups. This was, however, not the case for a2, when preference for H3N2/09 was significantly decreased or lost. Therefore, according to our observations, for all age groups total IgG titers were highest against recently circulating strains in both assessments, as opposed to VN antibody titers, which seemed to be highest against imprinting strains or strains encountered frequently in the past.

**Figure 5:**
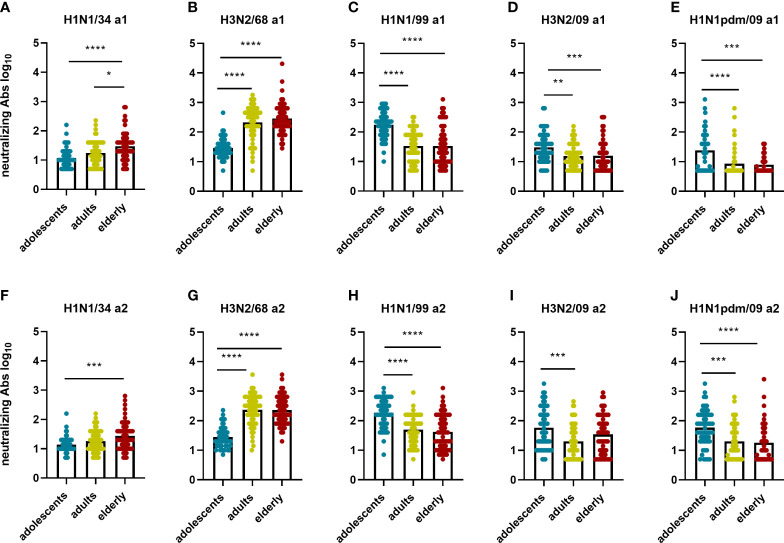
Birth year-related differences in neutralizing antibody titers per virus strain. The neutralizing antibody titers measured in the different age groups for each of the 5 influenza virus strains at a1 **(A–E)** and a2 **(F–J)** are displayed. Data were analyzed using Kruskal Wallis test followed by Dunn’s post-hoc test for multiple comparison. *P≤0.05; **P≤0.01; ***P≤0.001; ****P≤0.0001.

**Figure 6:**
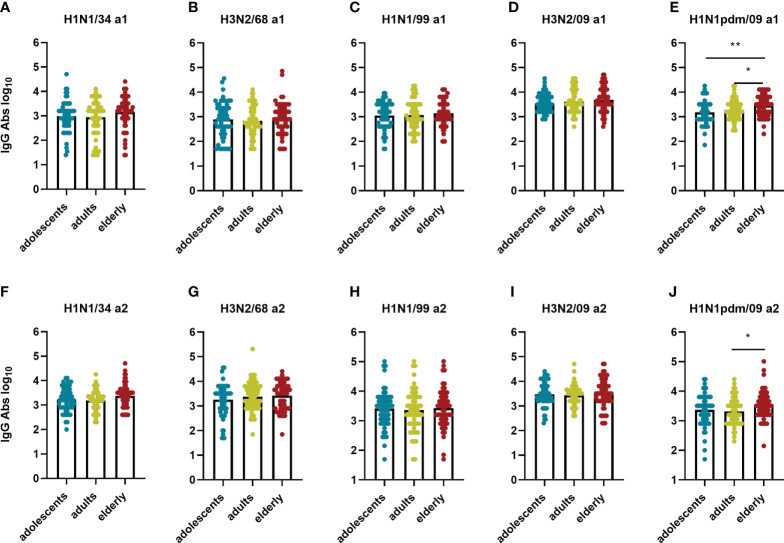
Birth year-related differences in IgG titers per virus strain. IgG titers measured in the different age groups for each of the 5 influenza virus strains at a1 **(A–E)** and a2 **(F–J)** are displayed. Data were analyzed using Kruskal Wallis test followed by Dunn’s post-hoc test for multiple comparison. *P≤0.05; **P≤0.01.

In order to study whether the immune response against a given IAV virus strain differs according to the birth year of the subjects, we compared neutralizing antibody titers (**Figure 5**) and total IgG titers (**Figure 6**) for every IAV strain included in this study across the different age cohorts. In both assessments, for the strain H1N1/34 we observed the highest levels of VN antibodies in the elderly cohort, for the H3N2/68 strain we observed them in the adults and elderly cohorts, while for H1N1/99, H3N2/09 and H1N1pdm09 we observed the highest VN antibody titers in the adolescents age cohort. Thus, for a given strain, VN titers were highest in the age group imprinted with this (or a closely related) strain (elderly for H1N1/34, adults for H3N2/68, adolescents for H1N1/99). Similarly, for a given strain the percentage of individuals with VN antibody titers ≥ 80 was highest for that age group which encountered the strain early in life (**Supplementary Figures 1 A–C**). Yet, for the elderly the percentage of individuals with VN titers ≥80 was substantially higher for H3N2/68 than for H1N1/34 despite the fact that the latter would be expected to be more closely related to the imprinting strain.

In contrast with the VN antibodies, IgG antibody titers did not show an age-related pattern for any of the strains with one exception: for the H1N1pdm09 strain we observed higher IgG titers in the elderly age group than in the other age groups (**Figure 6**). Interestingly, this was the case even at a1 when the elderly had not yet encountered the pandemic strain while the adolescents might have (the a1 samples of the adult and the elderly cohorts were taken in 2009 before the start of the pandemic, the a1 samples of the adolescents in 2011). This indicates the presence of substantial amounts of virus-binding antibodies cross-reactive with H1N1 pdm09 in the adult and especially in the elderly age group prior to the 2009 pandemic.

### Neutralizing antibody titers but not IgG antibody titers against the H1N1 pdm09 increased in-between the two assessments for all age cohorts

**Figure 7:**
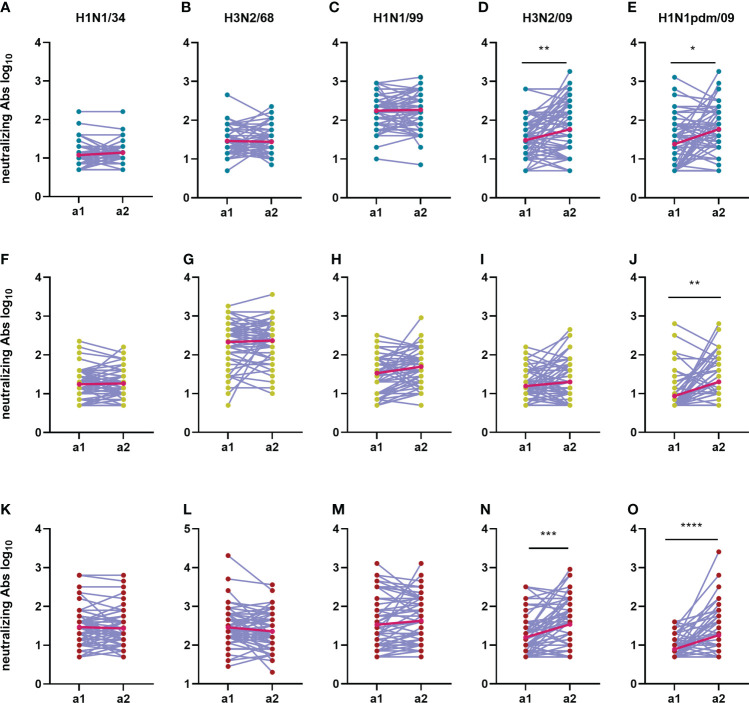
Change in neutralizing antibody titers over time during an interval of 5 years. The neutralizing antibody titers from 180 subjects during the first sampling moment (a1) are compared with the neutralizing titers from the second sampling moment (a2) for each virus strain (**A, F, K**: H1N1/34; **B, G, L**: H3N2/68; **C, H, M**: H1N1/99; **D, I, N**: H3N2/09; **E, J, O**: H1N1pdm/09) and age group (**A–E**: adolescents; **F–J**: adults; **K–O**: elderly). Titers of a given individual in a1 and a2 are connected by a line. Geometric means for the two assessments are depicted in the graphs as bright red dots which are connected by a red line for easy visualization of the mean increase. Data were compared using Wilcoxon matched-pairs signed rank test. *P≤0.05; **P≤0.01; ***P≤0.001; ****P≤0.0001.

**Figure 8:**
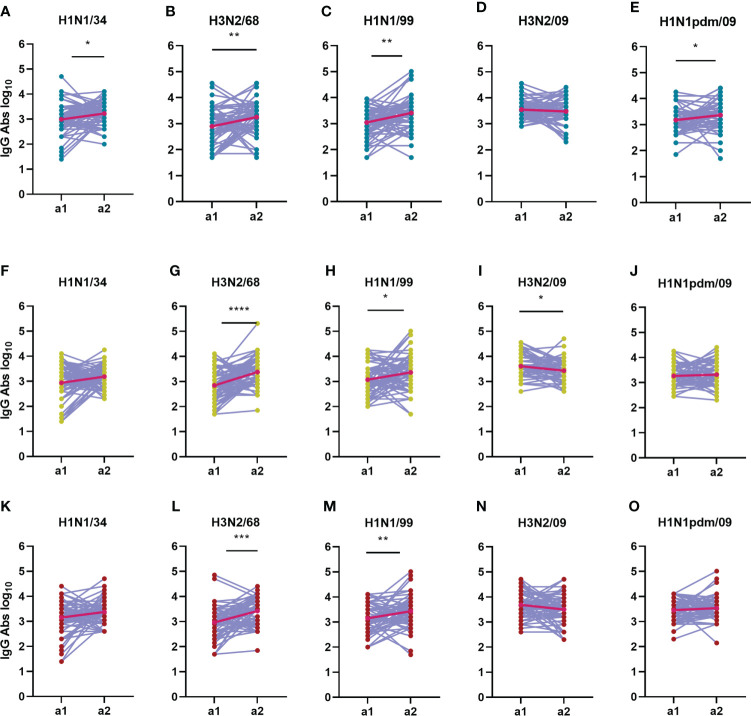
Change in total IgG antibody titers over time during an interval of 5 years. The IgG antibody titers from 180 subjects during a1 are compared with the IgG titers from a2 for each virus strain (**A, F, K**: H1N1/34; **B, G, L**: H3N2/68; **C, H, M**: H1N1/99; **D, I, N**: H3N2/09; **E, J, O**: H1N1pdm/09) and age group (**A–E**: adolescents; **F–J**: adults; **K–O**: elderly). Titers of a given individual in a1 and a2 are connected by a line. Geometric means for the two assessments are depicted in the graphs as bright red dots connected by a red line. Data were compared using Wilcoxon matched-pairs signed rank test. *P≤0.05; **P≤0.01; ***P≤0.001.

An important aim of our study was to investigate how imprinting with and sequential exposure to different IAV virus strains affects the humoral immune response to a novel pandemic IAV strain in subjects belonging to different age groups. To investigate this aspect, for each given age cohort we compared VN antibody titers and total IgG titers against all five tested IAV strains between assessment 1 and 2 (**Figures 7**, **8**). The increase of the average VN titers against H1N1pdm was significant in all age cohorts; the strongest increase was seen for the elderly cohort with an average net fold change between the 2 assessments of 8.3, against a net fold increase of 1.8 in the adults and 1.7 in the adolescents. VN antibodies specific for H3N2/09 also increased significantly between a1 and a2 in the adolescents and elderly cohorts but not in the adults possibly reflecting exposure by natural infection (adolescents) or vaccination (elderly) (**Figure 7**). In contrast, VN antibody titers to the older virus strains showed little changes over the 5-year period between a1 and a2. When analysing the VN antibody titers on an individual basis, we observed that most individuals had rather stable titers (less than 2-fold change) over the 5-year period for all viruses except for H1N1pdm (and H3N2/09 for the adolescent and elderly) for which a considerable percentage displayed increased titers (**Supplementary Figures 1D–F**). Increases in VN titers against H1N1pdm were not paralleled by increases in VN titers against imprinting H1N1 strains (H1N1/34 in the elderly, H1N1/99 in the adolescents) or the imprinting H3N2/68 strain in case of the adults. Thus, we did not find evidence for a boosting effect regarding VN antibodies to previously encountered viruses, not even if the priming virus was of the same virus subtype as the newly encountered virus. The 10 subjects with the highest titers to H1N1pdm09 in a2 and the 10 subjects with the lowest titers to this virus strain had very similar patterns of responses to the other virus strains (**Supplementary Figure 2**). These observations suggest that IAV-specific pre-existing immunity did not affect the induction of VN antibodies to H1N1pdm09.

As far as influenza specific IgG is concerned, we observed that IgG titers appeared to be very dynamic in time on an individual base, increasing for some subjects while decreasing for others over the 5-year interval (**Figure 8** and **Supplementary Figures 1G–I**). This phenomenon was observed in all age groups. The strongest average increase in IgG antibodies was seen against H3N2/68 in the adults and elderly age groups, with a net fold change of 4.5 and 1.5, respectively (**Figure 8**). For the adolescents, the strongest increase in IgG titers was seen against H1N1/99, with a net fold change of 4 (**Figure 8**). Interestingly, there was no significant increase in IgG antibodies to H1N1pdm09 in the adults and elderly between a1 and a2, showing once again that IgG titers do not seem to mirror neutralizing titers. Nevertheless, the number of individuals with an increase in IgG titer during or shortly after introduction of the novel H1N1pdm virus was higher for H1N1 viruses than for H3N2 viruses, and was highest against the oldest H1N1 viruses in the panel.

Taken together, our data show that neutralizing antibody titers increased significantly against recently encountered strains, while IgG antibody titers increased significantly against strains encountered in the past for all age groups in between the 2 assessments.

### NA inhibiting antibodies against N1 from H1N1pdm09 differ in magnitude between age groups and increased after the advent of H1N1pdm09

**Figure 9:**
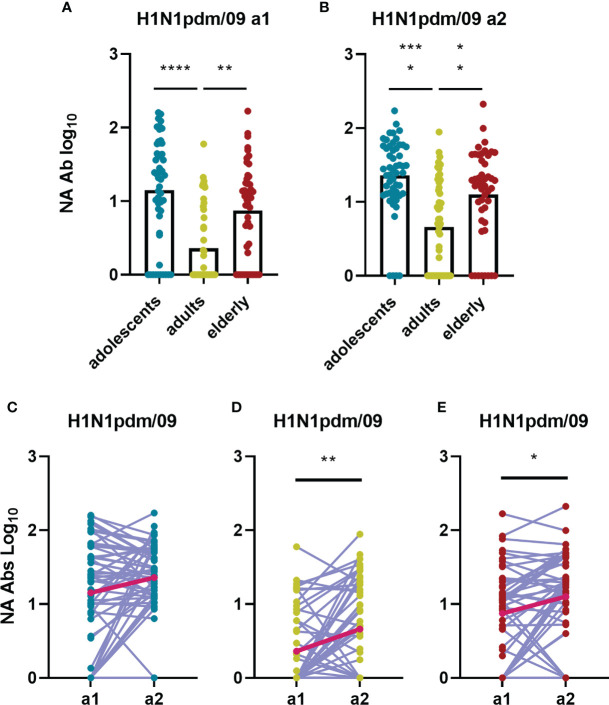
Birth year-related differences in influenza H1N1pdm09 specific NA antibody titers and how these change over time during a 5-years interval. Antibodies inhibiting the NA of the H1N1pdm09 strain were determined by ELLA and are depicted for the different age groups for assessment 1 **(A)** and assessment 2 **(B)**. Changes in NA-inhibiting antibody levels between a1 and a2 are indicated for adolescents [**(C)** n=53], adults [**(D)** n=48] and elderly [**(E)** n=47]. In **(A, B)** data were analyzed using Kruskal Wallis test followed by Dunn’s post-hoc test for multiple comparison. In (**C, D**) and E Wilcoxon matched-pairs signed rank test was performed to show significant differences. GM is indicated with a red line in **(C–E)**. *P≤0.05; **P≤0.01; ***P≤0.001; ****P≤0.0001.

The second major surface glycoprotein of influenza virus, NA, has enzymatic sialidase activity which is important for the release of virions from mucins and for the release of budding virus from infected cells. Inhibition of this sialidase activity is a very important mechanism of action of NA-specific antibodies (22). Using the ELLA assay, we quantified NA inhibiting antibodies against neuraminidase from H1N1pdm09 (**Figure 9**). Interestingly, anti-N1 antibodies were found to be significantly higher in adolescents and elderly than in adults (**Figures 9A, B**). High titers were expected in the adolescent population since a rather large fraction of this group had already seen the virus in question, having been sampled for a1 after 2009 (in 2011). The unexpectedly high levels of anti-N1 in the elderly population prior to the H1N1 2009 pandemic were likely caused by a previous encounter with an antigenically similar virus early in life. Most probably this was not the H1N1 virus circulating since 1977 as this was also frequently seen by the adult population which displayed rather low levels of NA inhibiting antibodies at a1. For the adults and elderly, who both encountered the H1N1pdm09 virus for the first time between a1 and a2, we observed a significant increase in NA inhibiting antibodies over the 5-year sampling interval, with the stronger increase seen in the adult cohort (fold change=2.1, **Figures 9C–E**).

### Absence of sex-related differences in IgG and neutralizing antibody titers

Since it was recently described that the level of humoral immunity might differ between the sexes, we next compared influenza-specific antibody titers between male and female individuals. However, for none of the influenza virus strains studied we could find any sex-related differences in VN or IgG antibody titers. Both these antibody types were comparable in both sexes in both assessments and changed in a comparable way over the 5-year time period investigated (**Supplementary Figures 3A, B**). Similarly, we could not detect any sex-related differences for NA-specific antibody titers or changes in these titers (**Supplementary Figure 3C**).

## Discussion

In this study, we sought to get a better understanding of how natural infection (and vaccination) induces and shapes broad and long-lived immune responses to influenza virus. To this end, we determined levels of VN and virus-binding antibodies to five strains of IAV in young, adult and elderly individuals at two timepoints five years apart. We observed that (i) levels of neutralizing antibodies were highly strain- and age-dependent and predominantly directed to virus strains encountered in the past, (ii) levels of virus-binding IgG did not show age-related differences and were highest against recently encountered strains, (iii) the largest changes in neutralizing antibody levels occurred against recent strains while the largest changes in virus-binding IgG levels were towards older strains. Our results demonstrate that VN and virus-binding antibodies follow different ‘evolutionary’ pathways which are – to a certain extent - compatible with the antigenic seniority theory.

The first aim of our study was to investigate in how far titers of VN and virus-binding antibodies in our study group displayed an age-dependent preference to a certain IAV strain. Several epidemiological studies describe that VN antibody titers to different IAV strains are dependent on birth year of the subjects (6, 7, 23–25). This phenomenon has been ascribed to immune imprinting by the very first encountered IAV strain (26). Imprinting happens very early in life, as most children by the age of 3 have already been infected at least once with influenza virus (10). We observed that for adolescents and adults VN titers were indeed highest against the presumable imprinting strain, H1N1/09 and H3N2/68, respectively. However, the elderly cohort showed the highest neutralizing titers against H3N2/68, rather than against H1N1/34 which should be the virus more closely related to the first IAV strain this age group encountered. A possible explanation is that the imprinting strain for this age group, born 1942-1947, was in fact the influenza A’ strain that emerged in 1946 and, although being classified as belonging to the H1N1 subtype, is antigenically very distant from H1N1/34 (27). Alternatively, the appearance of H2N2 in 1956 and of H3N2 in 1968 and frequent boosting of H3N2-specific antibodies in the subsequent decades due to long term circulation of this subtype might have resulted in a ‘reset’ of the original strain preference. Future studies should investigate the antibody titers to the A’ strain and H2N2 virus which was beyond the scope of this study. Regarding virus-binding IgG we did not observe age-dependent strain preferences. In fact, the IgG titers measured were rather similar across age groups and were somewhat higher against recent than against past strains. Our results are in line with those of Meade et al. who also found IgG against a range of different IAV strains and somewhat stronger reactivity against more recent strains (16). Interestingly, though never exposed to H1N1/34 and H3N2/68, adolescents had similarly high titers to these strains as elderly and adults. Along the same line, adults and elderly had substantial amounts of IgG recognizing H1N1pdm even before the 2009 pandemic. These observations imply that virus-binding IgG is highly cross-reactive. This is important as it has recently been described that the level of virus-binding but not necessarily neutralizing antibodies is an independent predictor of protection against influenza (28). It should be noted that we used inactivated whole influenza virus for coating of the ELISA plates since we were interested in the total antibody reactivity to IAV rather than to HA alone. Nevertheless, our results are very similar to those of previous studies which mostly focused exclusively on HA-specific immunity (23).

The second aim of our work was to understand how pre-existing immunity, shaped by previous virus encounters, affected the immune response to a novel IAV strain and vice versa how exposure to the novel strain impacted on antibody titers to older strains. In all age groups, IgG endpoint titers against H1N1pdm09 were quite similar at the two assessments. In contrast, neutralizing antibodies to this virus strain increased significantly in most of the subjects in all age cohorts, and in particular in the elderly cohort indicating that antibodies to novel epitopes were effectively induced irrespective of pre-existing immunity but represented only a minor fraction of the total influenza virus-specific antibody response. The particularly high responses observed in elderly at a2 could be due to H1N1pdm09 vaccination in late 2009 and in subsequent years as influenza vaccination is recommended for this age group. Another explanation might be that the elderly cohort was imprinted with an H1N1 strain antigenically very similar to H1N1pdm09 and the H1N1pdm09 strain thus activated memory B cells (26). However, in a study assessing responses to H1N1pm09 vaccine in health care workers exposed to different IAV strains during early childhood Madsen et al. did not find evidence for an effect of H1N1 imprinting (29). With regard to the effect of exposure to the new virus strain on antibody titers to previously encountered strains we observed that IgG titers against past strains were boosted for most of the subjects during the 5-year interval, in particular IgG titers against H1N1 virus strains. These observations are in line with previous data showing that the encounter with an IAV pandemic strain can boost antibodies targeting conserved viral epitopes, which are shared among strains and sometimes even among influenza phylogenetic groups (30). In contrast, levels of VN antibodies to older strains did not change significantly and few individuals showed more than 2-fold increases in titer.

To get further insight in pre-existing immunity and response to a new virus type we also investigated the antibody response to H1N1pdm09 neuraminidase. NA is the second major surface glycoprotein of influenza virus and it has enzymatic sialidase activity which is important for the release of virions from mucins during viral infection and for the release of budding virus from infected cells (29, 31–33). NA-specific antibodies inhibit this sialidase activity and can thereby confer protection from influenza virus infection in humans (29, 31, 34). In our study, NA-inhibiting antibodies against N1 from H1N1pdm09 were lowest in the adult cohort, the only cohort imprinted with an H3N2 IAV. This result suggests that an imprinting effect not only holds true for HA but also for NA as also suggested by others (32). After the advent of the H1N1pdm09 virus NA-inhibiting antibodies increased significantly, more for the adults than for the other age groups.

From the observations regarding antibody titers to HA as well as NA we can conclude that neither the existence of influenza immune memory nor the boosting of pre-existing IgG antibodies to past strains, formed an obstacle for the generation of a successful neutralizing immune response to a novel influenza pandemic strain.

The final aim of our study was to investigate whether our data were in line with the original antigenic sin (OAS) theory or the antigenic seniority (AS) theory (10, 17, 23, 26, 28, 32, 34–37). OAS is believed to be caused mainly by epitope masking which prevents activation of naïve B cells optimally fitting the new epitopes, but not previously activated B cells. In contrast, the AS theory relies mainly on the fact that activation of memory B cells is more easily achieved than activation of naïve ones (10). In our study, the VN antibody titers roughly followed the AS theory: titers were highest against the imprinting strain (except for the elderly) but, in line with the AS theory, the presence of these antibodies did not impair effective induction of neutralizing antibodies to the newly encountered H1N1pdm09 strain. However, in contrast to what the AS theory predicts, the encounter of the new H1N1pdm strain did not result in further increase of neutralizing antibody titers to the earlier encountered strains, not even earlier encountered H1N1 viruses. The IgG titers, on the other hand, were highest against recent virus strains and not against the imprinting strain as predicted by both the OAS and the AS theory. Yet, encounter of the H1N1pdm09 strain did not result in effective induction of IgG to this strain, in agreement with the OAS theory. Rather, IgG titers to several earlier encountered strains were boosted, the latter in line with the AS theory. Thus, kinetics of antibodies to influenza virus depend on the type of antibodies studied, rather strain specific VN antibodies or more cross-reactive virus-specific IgG, and are generally more complex than predicted by the current theories. The debate is open as to whether VN or ELISA binding assays best recapitulate the picture of influenza-specific humoral immunity in humans in general (17, 24, 38). We believe that, for a comprehensive analysis of the influenza antibody landscapes in humans, both types of antibodies are to be taken into account, as they both can contribute to protection but do so in different ways (4, 28).

The strength of our study is that it involved rather large groups of individuals from three different birth cohorts and provides data on binding as well as VN antibodies to 5 different influenza virus strains, covering the H1N1 as well as the H3N2 subtype. Using serum samples from the same individuals taken 5 years apart, allowed us to assess the dynamics of the antibody responses to these virus strains, for the adults and elderly even over a period which saw the arrival of the novel H1N1pdm09 strain. However, our study also has some limitations. Firstly, we were not able to obtain data regarding influenza infection or vaccination history for any of the subjects. However, since we were interested in a generic humoral immune response generated by any encounter with IAV, the nature of the encounter was, in our study, of minor importance. Secondly, as a representative of early H1N1 strains we used A/PR/8/34 rather than a virus of the A’ sublineage which would have been more closely related to the first virus encountered by our elderly cohort. Thirdly, the time for the two assessments was different in adults and elderly compared with adolescents; for this reason, we were able to assess “pre” and “post” pandemic serum samples only for the adults and elderly cohorts. Fourthly, for practical reasons we used egg-grown virus which bears the risk of having changed from the original isolate due to adaptation to growth in chicken cells. However, since we were mainly interested in differences in antibody reactivity among the age groups and ran all assays for all age groups with the same virus batches, we considered the possible problem of antigenic change of minor importance. Finally, though bigger than most cohorts so far investigated, our study cohort contained only 180 individuals, 60 per age group. While this population size is more than appropriate to perform statistics on the data, new longitudinal studies are needed to further confirm our findings in the future; these studies should ideally include a higher number of subjects, which should be followed for a longer period of time. Moreover, a further expansion of the panel of viruses would be desirable.

The findings of our study suggest that IAV immune imprinting and immune memory are not an obstacle for the generation of a successful neutralizing immune response to a novel pandemic influenza virus strain. The observed landscape of IAV-specific neutralizing antibodies supports the AS theory, suggesting that influenza virus specific immunity tends to be strongest towards imprinting strains and is inclined to be broadened with every new influenza virus encounter both by infection or vaccination. Yet, IAV-binding antibodies did not follow this line and were found to be strongest against recently encountered strains. Our study contributes to unravelling influenza specific immune memory and its evolution during life and thus to knowledge needed to allow for rational design of better and smarter vaccination strategies in the future.

## Data availability statement

The raw data supporting the conclusions of this article will be made available by the authors, without undue reservation.

## Author contributions

FS, FZ, RC, and AH conceptualized the study. FS, ES, and AJ performed the experiments and analyzed the data. FS and AH wrote the draft manuscript. FZ and RC revised the manuscript. All authors read the manuscript and approved submission.

## Funding

FS was supported by the EU Horizon 2020 Program under the Marie Skłodowska-Curie grant agreement 713660 (Pronkjewail) and received a grant from the Gratama Foundation, Groningen.

## Acknowledgments

The Lifelines Biobank initiative has been made possible by subsidy from the Dutch Ministry of Health, Welfare and Sport, the Dutch Ministry of Economic Affairs, the University Medical Center Groningen, University of Groningen and the Northern Provinces of the Netherlands. The authors wish to acknowledge the services of the Lifelines Cohort Study, the contributing research centres delivering data to Lifelines, and all the study participants. The authors wish to thank Stijn de Vos (Department of PharmacoTherapy, Epidemiology & Economics, Groningen Research Institute of Pharmacy, University of Groningen, Groningen, The Netherlands) for help with the statistical analysis in this work.

## Conflict of interest

The authors declare that the research was conducted in the absence of any commercial or financial relationships that could be construed as a potential conflict of interest.

## Publisher’s note

All claims expressed in this article are solely those of the authors and do not necessarily represent those of their affiliated organizations, or those of the publisher, the editors and the reviewers. Any product that may be evaluated in this article, or claim that may be made by its manufacturer, is not guaranteed or endorsed by the publisher.
