# Supplementary material for: The evolution of humoral immune responses to past and novel influenza virus strains gives evidence for antigenic seniority
Source: Front Immunol. 2022 Sep 2;13:987984. doi: 10.3389/fimmu.2022.987984 (PMC9478913; doi:10.3389/fimmu.2022.987984)
Supplement: Supplementary file 1 [file DataSheet_1.docx]

Supplementary Material

## Supplementary Figure 1

##

**Supplementary Figure 1: Virus strain dominance and changes by number of individuals per age cohort.** For adolescents (A, D, G), adults (B, E, H), and elderly (C, F, I) the number of study participants with a VN antibody titer ≥80 (A-C) and the number of participants with increasing, stable, or decreasing MN titers (D-F) and IgG titers (G-H) between a1 and a2 are depicted. A-C: dark bars = a1, light bars =a2. D-I: dotted bars = increasing, hatched bars = stable, plain bars = decreasing titers.

**Supplementary Figure 2**

**Supplementary Figure 2. Antibody profiles of individuals across different influenza virus strains.** For the 10 sera with the highest (A, B, C) and the 10 sera with the lowest (D, E, F) neutralizing antibody titers to H1N1pdm/09 at a2 (open symbols) the neutralizing antibody titers at a1 are depicted for the different virus strains studied (closed symbols) and the different age groups, children (A, D), adults B, E) and elderly (C, F).

**Supplementary Figure 3**

**Supplementary Figure 3: Sex-related differences in influenza-specific antibody titers.** The VN (A) and IgG antibody titers (B) (n=180) at a1 and a2 are displayed to allow comparison between males and females for each virus strain in both assessments. Titers of antibodies inhibiting the NA of H1N1pdm09 (n=148) samples per sex and assessment are displayed in C. Data were analyzed using Kruskal Wallis test followed by Dunn’s post-hoc test for multiple comparisons.
